# Musik und Bewegung als therapeutische Ressourcen bei Demenz

**DOI:** 10.1007/s00108-022-01445-2

**Published:** 2022-12-01

**Authors:** Reto W. Kressig

**Affiliations:** 1 Universitäre Altersmedizin FELIX PLATTER, Burgfelderstr. 101, 4055 Basel, Schweiz; 2 Universität Basel, Basel, Schweiz

**Keywords:** Musiktherapie, Bewegung, Alzheimer-Erkrankung, Tanztherapie, Musikalisches Gedächtnis, Music therapy, Exercise, Alzheimerʼs disease, Eurhythmics, Musical memory

## Abstract

In der Betreuung von Menschen mit Demenz spielen Musik und Bewegung seit Jahrzenten eine wichtige Rolle. Die dazu bestehende wissenschaftliche Evidenz ist immer noch relativ klein, wächst aber. Der erst seit knapp zehn Jahren bekannte Umstand, dass das musikalische Gedächtnis bis in fortgeschrittene Demenzstadien intakt bleibt, hat Musik bei Hirnleistungsabbau zunehmend zur therapeutisch genutzten Ressource werden lassen. So können gesungene Texte viel besser erinnert werden als gesprochene. Die gezielte musikalische Stimulierung der frontalen Hirnregionen hat zudem häufig positive Wirkungen auf demenzassoziierte Verhaltensauffälligkeiten. Bewegung kombiniert mit Musik in Form von Tanz oder Rhythmik scheint neben positiven kognitiven Effekten auch mit Verbesserungen des Gleichgewichts und der Gangsicherheit einherzugehen.

## Abstract (en)

Music and exercise have played a major role in the care of people with dementia for decades. The scientific evidence for such interventions is still relatively scarce but growing. The fact which has been known for nearly 10 years that brain regions associated with musical long-term memory remain intact up to advanced stages of dementia made music a major therapeutic resource in cognitive decline. Therefore, sung texts are learned and recalled better than spoken texts. Furthermore, the specific music-related stimulation of frontal brain regions has frequently shown positive effects on conspicuous behavioral disorders associated with dementia. Exercise in combination with music, in the form of dance or eurhythmics, not only seems to positively affect cognition but also balance and gait safety among patients with dementia.

Nichtmedikamentöse Therapieformen bei Demenz haben im Vergleich zu medikamentösen Therapien den großen Vorteil fehlender medikamentöser Interaktionen und Nebenwirkungen. Allerdings sind sie organisatorisch wie auch hinsichtlich der Finanzierung aufwendiger in der Umsetzung. Trotzdem bieten mittlerweile viele spezialisierte Betreuungsinstitutionen für Patienten mit Demenz solche Interventionen an, was auch das wissenschaftliche Interesse an deren Wirkungsmechanismen hat wachsen lassen. Musikalische Interventionen haben hier ein besonders hohes Potenzial, da sie auf ein – trotz fortgeschrittener Demenz – weitgehend intaktes musikalisches Gedächtnis zurückgreifen können.

Die publizierte wissenschaftliche Evidenz zur therapeutischen Bedeutung von Musik bei demenziellen Erkrankungen ist im Vergleich zu medikamentösen Therapieansätzen bei Demenz gering. Trotzdem: Gab es zum Thema Musik und Demenz im Jahr 2000 noch insgesamt 16 wissenschaftliche Beiträge pro Jahr (PubMed: Suchworte „music“ und „dementia“), ist deren Anzahl im Jahr 2021 auf 123 angestiegen. Leider sind davon nur 9 Publikationen als „klinische Studien“ klassiert, was die Hauptherausforderung in dieser „jenseits des Mainstreams“ gelegenen Thematik unterstreicht: Planung und Durchführung guter randomisierter, kontrollierter Interventionsstudien sind extrem anspruchsvoll. Die beteiligten Wissenschaftler kämpfen nicht selten mit

- Problemen der Rekrutierung (aufwendige Einholung der Studieneinverständniserklärung),
- fragilen und symptomatisch heterogenen Studienteilnehmern,
- komplexen methodologischen Fragestellungen (Wahl sinnvoller quantitativer und qualitativer Endpunkte) und letztlich
- einer schwer zu findenden Finanzierung.

Trotz der wissenschaftlich kargen Beweisführung gibt es viele auf Demenz spezialisierte Institutionen, die Musik in irgendeiner Form (meist in Kombination mit Bewegung) in den Betreuungsalltag integriert haben und immer wieder von positiven therapeutischen Erlebnissen berichten.

## Was sagt die Wissenschaft?

In einer kürzlich veröffentlichten randomisierten, kontrollierten spanischen Interventionsstudie [1] unter Einschluss von 90 Pflegeheimbewohnern mit leichter bis mittelschwerer Alzheimer-Erkrankung wurde eine aktive musikalische Gruppenintervention mit passivem Musikhören (ebenfalls im Gruppensetting) verglichen – als Kontrolle diente eine Gruppe ohne Musikintervention. Die aktive Musikgruppenintervention bestand in einem Willkommenslied, Rhythmik, Tanzen, einem Musikquiz und einem Abschiedslied. Die Musikhörgruppe bekam in sitzender Position Musikaufzeichnungen aus dem Computer zu hören, wobei jeweils Sänger wie Titel der gespielten Musik vom Gruppenanimator bekannt gegeben wurden und auch die Möglichkeit für die Pflegeheimbewohner bestand, ihre Erinnerungen und Gefühle zur gehörten Musik auszudrücken und zu diskutieren. Die Wahl der gespielten Musikstücke wurde mit den vorher mittels Fragebogen ermittelten Musikpräferenzen der Studienteilnehmer abgestimmt. Der Kontrollgruppe wurden dokumentarische Naturvideos gezeigt, die vor allem von der afrikanischen Tierwelt handelten und akustisch lediglich Naturgeräusche und keine Musik beinhalteten. Jede Intervention dauerte rund 45 min und fand 2‑mal wöchentlich über 3 Monate statt. Im Vergleich zur Kontrollintervention vermochte die aktive Musikintervention die Symptome von drei Hirndomänen zu verbessern (Kognition, Verhalten, funktioneller Status), das passive Musikhören zeigte lediglich stabilisierende Effekte auf neuropsychiatrische Symptome.

Eine ebenfalls kürzlich veröffentlichte systematische Übersicht und Metaanalyse [2] untersuchte die Effekte von aktivem Musikmachen bei Patienten mit leichter kognitiver Beeinträchtigung und Demenz. Dabei wurden 21 randomisierte, kontrollierte Studien mit insgesamt 1472 Teilnehmern in die Analyse eingeschlossen. Alle Studien nutzten entweder die Reproduktion von Musik mit Singen bzw. Spielen eines Musikinstruments oder Musikimprovisation aus dem Moment heraus. Über alle Studien zeigte die Musikintervention einen kleinen, aber signifikanten positiven Effekt auf die Kognition der Studienteilnehmer. In einzelnen Studien fanden sich auch positive Effekte auf Stimmung und Lebensqualität.

## Musik, musikalisches Gedächtnis und Alzheimer-Erkrankung

**Figure 1:**
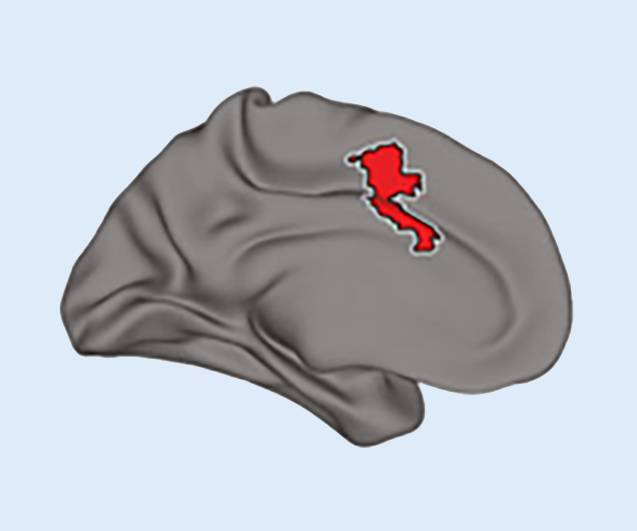

Jacobsen et al. [3] zeigten im Jahr 2015 eindrücklich auf, dass das Hirnareal des musikalischen Langzeitgedächtnisses (Abb. 1) im Verlauf einer Alzheimer-Erkrankung – verglichen mit dem restlichen Hirn – lediglich eine minimale kortikale Atrophie und Disruption des Glukosemetabolismus aufweist. Dies erklärt die immer wieder gemachte klinische Beobachtung, dass Patienten in fortgeschrittenen Demenzstadien mit Sprachverlust beim Hören von bekannten Liedmelodien fehlerfrei ganze Liedstrophen mitsingen können.

Gemäß einer Interventionsstudie kann Musik das verbale Gedächtnis bei Alzheimer-Erkrankung stärken

Dass Musik das verbale Gedächtnis von Patienten mit Alzheimer-Erkrankung stärken kann, wurde in einer anderen, im selben Jahr publizierten Interventionsstudie [4] gezeigt. Kognitiv Gesunde wie auch Menschen mit leichter Alzheimer-Demenz konnten sich an gesungene Texte im Vergleich zu den gleichen, aber gesprochenen Texten signifikant besser erinnern. Dreimonatige musikalische Gruppeninterventionen bei Patienten mit früher Alzheimer-Erkrankung führten unmittelbar und auch 6 Monate nach Intervention zu signifikanten kognitiven, emotionalen und sozialen Verbesserungen [5]. Fünf Monate mit individueller Musikintervention bei Patienten mit Demenz in fortgeschritteneren Stadien erbrachten eine signifikante Reduktion neuropsychiatrischer Symptome und verbesserten das beobachtete Wohlbefinden der Patienten [6].

## Bewegung und rein körperliche Aktivitäten zur Behandlung von Verhaltensauffälligkeiten bei Alzheimer-Demenz

Die größte Anzahl nichtpharmakologischer Studien zur Behandlung von demenzassoziierten Verhaltensauffälligkeiten wurde mit körperlichen Aktivitätsinterventionen durchgeführt [7]. Obwohl diese Studien sehr heterogen bezüglich Typ und Stadium der Demenz, aber auch hinsichtlich Art und Dauer der körperlichen Aktivität waren, zeigten praktisch alle einen positiven Effekt auf demenzassoziierte Verhaltensauffälligkeiten. Die vermuteten Wirkungsmechanismen sind spannend und vielfältig, bedürfen aber sicherlich noch zusätzlicher Forschung. Postuliert werden psychologische (beispielsweise verbesserter Schlaf und Stressreduktion) und neurobiologische Mechanismen (Veränderung der Neurotransmitterkonzentrationen, gesteigerte Neurotrophinsynthese, Stimulierung des Immunsystems).

Ein regelmäßiges und intensives körperliches Training bei zu Hause lebenden Patienten mit Alzheimer-Erkrankung kann aber auch wesentliche funktionelle Verbesserungen bewirken. Bei 2‑mal wöchentlichem Training über ein Jahr wurden in der finnischen FINALEX-Studie [8] gegenüber der Kontrollgruppe nicht nur funktionelle Vorteile, sondern auch eine geringere Sturzrate gezeigt.

Aber auch deutlich weniger intensive, 1‑mal wöchentlich während 10 Monaten durchgeführte biografisch inspirierte körperliche Aktivitäten können die vielfach sehr störende Apathie von Demenzkranken verbessern; allerdings nur so lange, wie die Aktivitäten auch durchgeführt werden [9].

Aus klinischer Erfahrung hängt die Compliancerate für körperliche Aktivitäten bei Patienten mit Demenz, ähnlich wie bei kognitiv gesunden Senioren, von mehreren Zusatzfaktoren ab. Entscheidend für die Nachhaltigkeit eines körperlichen Interventionsprogramms sind

- gute Zugänglichkeit des Trainingsorts,
- angepasste Zeitwahl (Wochentag, Tageszeit),
- vernünftige Trainingsintensität,
- Möglichkeiten des sozialen Austauschs,
- Involvierung der Betreuer und
- aktive Empfehlungsunterstützung der Trainingsaktivität durch den Hausarzt und andere Schlüsselpersonen.

## Musik, Rhythmus und Gangmotorik

**Figure 2:**
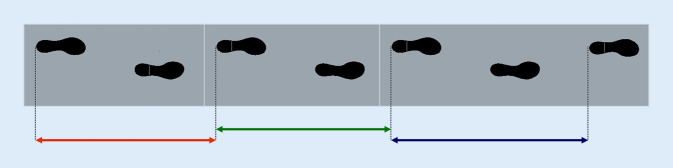

**Figure 3:**
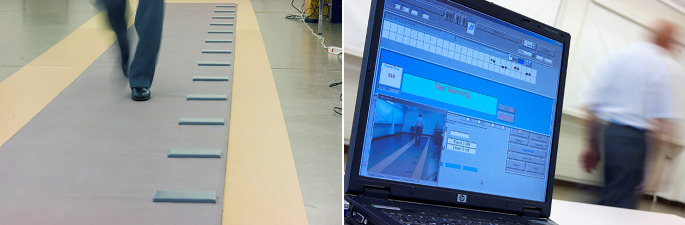

Anders als andere Lebewesen muss der Mensch das aufrechte Gehen in den ersten Lebensjahren erlernen. Erst mit Erreichen des jungen Erwachsenenalters hat sich das Gangbild und vor allem die Gangregelmäßigkeit (Abb. 2) in einem langen Lernprozess derart perfektioniert, dass die Schrittlängen von einem Schritt zum nächsten nahezu identisch sind. Mithilfe von für das klinische Setting erhältlichen Ganganalysesystemen kann die Gangvariabilität rasch und einfach ermittelt werden (Abb. 3). Ältere Menschen, die diese hohe Gangregelmäßigkeit verlieren, haben ein deutlich erhöhtes Sturzrisiko. Schritt-zu-Schritt-Längendifferenzen von weniger als 2 cm können dabei das Sturzrisiko bereits verdoppeln [10]. Feinste Gangunregelmäßigkeiten können aber auch Vorboten einer späteren Demenzerkrankung sein. In der Einstein-Aging-Kohortenstudie zeigten Kohortenteilnehmer mit frisch diagnostizierter Demenzerkrankung – im Vergleich zu kognitiv gesund gebliebenen Teilnehmern – bereits fünf Jahre früher eine signifikant erhöhte Variabilität der Schwingphase im Gangbild [11]. Mit fortschreitendem Demenzstadium erhöht sich die Gangunregelmäßigkeit und damit das Sturzrisiko kontinuierlich [12].

Bei motorisch-kognitiver „frailty“ mit entsprechend verminderten physiologischen Reserven kann ein imminentes Sturz- und Demenzrisiko mittels Gangtestung und Dual-task-Aufgabe demaskiert werden. Dabei muss der Patient während des Gehens mit Gangmessung gleichzeitig eine Arbeitsgedächtnisaufgabe lösen, beispielsweise eine Subtraktionsrechenaufgabe. Patienten mit einer deutlich erhöhten Gangunregelmäßigkeit unter „dual task“ haben ein erhöhtes Demenz- und Sturzrisiko [13, 14].

Feinste Gangunregelmäßigkeiten können Vorboten einer späteren Demenzerkrankung sein

Komplexe Hirnleistungen, wie Planen, Koordinieren, das Orchestrieren von Aktivitäten und die Entscheidung über Prioritäten (Aufmerksamkeit) bei Multitasking-Aktivitäten, sind Teil der zerebralen Exekutivfunktion, die im Frontalhirn lokalisiert ist [15]. Beim aktiven Musikhören spielt der präfrontale Kortex eine wichtige Rolle. Mittels funktioneller Bildgebung kann eine eindrückliche musikinduzierte Aktivierung im mediofrontalen Frontalkortex nachgewiesen werden [16]. Diese Arealassoziation erklärt die häufig gesehene und weiter oben beschriebene therapeutische Wirkung von Musik auf demenzassoziierte Verhaltensauffälligkeiten, deren Ursprung ebenfalls im Frontalhirn liegt.

## Musikbegleitete und -gesteuerte Bewegungsaktivitäten bei Demenz

**Figure 4:**
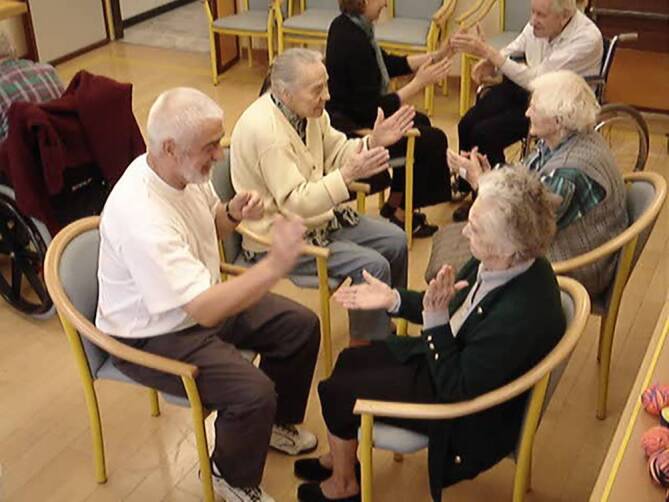

Spannend und auch immer wieder Gegenstand der Forschung ist die Hirnwirkung von mit Musik kombinierten Bewegungsaktivitäten wie Tanz und Rhythmik. In der Einstein-Aging-Kohortenstudie war regelmäßiges Tanzen als Freizeitbeschäftigung mit einem bis zu 80 % erniedrigten späteren Demenzrisiko assoziiert [17]. In einer Interventionsstudie mit Rhythmik nach Jaques-Dalcroze konnte das motorisch-kognitive Dual-task-Vermögen von zu Hause lebenden Senioren verbessert und das Sturzrisiko um über 50 % reduziert werden [18]. In fortgeschrittenen Demenzstadien scheint die Jaques-Dalcroze-Rhythmik neben der positiven Beeinflussung von „behavioral and psychological symptoms of dementia“ (BPSD) vor allem die sprachlichen Fähigkeiten zu fördern ([19]; Abb. 4). Im Gegensatz zu Rhythmik mit Kindern oder gesunden Erwachsenen gilt es bei der Durchführung von Rhythmikateliers mit Demenzkranken eine ganze Reihe von spezifischen Empfehlungen zu beachten (Infobox 1).

### Infobox Empfehlungen für die Durchführung von Rhythmikateliers bei Menschen mit fortgeschrittener Demenzerkrankung [20]

Empfehlungen für Rhythmik und andere Musik-Bewegungs-Interventionen bei älteren Patienten mit Demenz:

- Wahl *bekannter Melodien*
- Start mit *langsamen* Melodien und Rhythmen
- Genügend lange *Repetition* von Bewegungsabläufen (Automatisierung)
- Arbeit mit vornehmlich *visueller* und wenig verbaler Instruktion
- *Freiwillige Helfer* sitzen vor dem Patienten (Lernen im Spiegel)
- Gute Erfahrungen mit gemischten Gruppen (Patienten mit Demenz und kognitiv Gesunde)
- Anzahl freiwilliger Helfer basiert auf der Anzahl an Patienten mit fortgeschrittener Demenz
- Maximale sensorielle Stimulierung (Augen, Ohren, Berührung)
- Übungen mit möglicher sozialer Interaktion
- Komfortable lockere Kleidung, Schuhe mit dünner Sohle und gutem knöchelumfassendem Verschluss (guter sensorieller Halt)

## Fazit für die Praxis

- Nichtpharmakologische Interventionen bei Patienten mit Demenz sind ein wesentlicher Bestandteil des modernen Demenzmanagements.
- Die zu erwartende Hauptwirkung solcher Maßnahmen besteht in der positiven und nebenwirkungsfreien Beeinflussung von Verhaltensauffälligkeiten.
- Körperliche Aktivitätsprogramme zeigen zusätzliche Vorteile in Bezug auf Alltagsfunktionalität, Gleichgewicht und Gangsicherheit.
- Musik und musikbasierte Bewegungsprogramme wie Tanz und Rhythmik erscheinen besonders geeignet, noch vorhandene Hirnreserven zu mobilisieren und damit die Kognition signifikant zu verbessern.
